# Molecular Targets and Pathways Contributing to the Effects of Wenxin Keli on Atrial Fibrillation Based on a Network Pharmacology Approach

**DOI:** 10.1155/2020/8396484

**Published:** 2020-10-07

**Authors:** Yujie Zhang, Xiaolin Zhang, Xi Zhang, Yi Cai, Minghui Cheng, Chenghui Yan, Yaling Han

**Affiliations:** ^1^Liaoning University of Traditional Chinese Medicine, Liaoning, Shenyang 110847, China; ^2^Cardiovascular Research Institute and Department of Cardiology, The General Hospital of Northern Theatre Command, Liaoning, Shenyang 110840, China

## Abstract

**Background:**

Atrial fibrillation (AF) is the most common sustained arrhythmia and is associated with high rates of mortality and morbidity. The traditional Chinese medicine Wenxin Keli (WXKL) can effectively improve clinical symptoms and is safe for the treatment of AF. However, the active substances in WXKL and the molecular mechanisms underlying its effects on AF remain unclear. In this study, the bioactive compounds in WXKL, as well as their molecular targets and associated pathways, were evaluated by systems pharmacology.

**Materials and Methods:**

Chemical constituents and potential targets of WXKL were obtained via the Traditional Chinese Medicine Systems Pharmacology (TCMSP). The TTD, DrugBank, DisGeNET, and GeneCards databases were used to collect AF-related target genes. Based on common targets related to both AF and WXKL, a protein interaction network was generated using the STRING database. Gene ontology (GO) and Kyoto Encyclopedia of Genes and Genomes (KEGGs) pathway enrichment analyses were performed. Network diagrams of the active component-target and protein-protein interactions (PPIs) were constructed using Cytoscape.

**Results:**

A total of 30 active ingredients in WXKL and 219 putative target genes were screened, including 83 genes identified as therapeutic targets in AF; these overlapping genes were considered candidate targets for subsequent analyses. The effect of treating AF was mainly correlated with the regulation of target proteins, such as IL-6, TNF, AKT1, VEGFA, CXCL8, TP53, CCL2, MMP9, CASP3, and NOS3. GO and KEGG analyses revealed that these targets are associated with the inflammatory response, oxidative stress reaction, immune regulation, cardiac energy metabolism, serotonergic synapse, and other pathways.

**Conclusions:**

This study demonstrated the multicomponent, multitarget, and multichannel characteristics of WXKL, providing a basis for further studies of the mechanism underlying the beneficial effects of WXKL in AF.

## 1. Introduction

Atrial fibrillation (AF) is one of the most common arrhythmias, and its prevalence is increasing [1]. The occurrence of AF can lead to heart failure, embolism, and stroke and significantly increases the rates of disability and death, presenting a serious health issue [2, 3]. Therefore, the prevention and treatment of AF are major concerns.

According to the American Heart Association practice guidelines, current strategies for AF management include rate control, rhythm control, anticoagulation, lifestyle, and risk factor management [4]. Antiarrhythmic drugs and catheter ablation are the main treatment options. However, available medications for AF are suboptimal based on the high rate of arrhythmia recurrence and the potential proarrhythmic effect [5, 6]. Catheter ablation is also limited by a high rate of recurrence, and patients often require additional surgery [6, 7]. Therefore, researchers, clinicians, and patients seek new effective and safe treatment strategies for AF.

Traditional Chinese medicines (TCMs) have been used for the prevention and treatment of arrhythmias in China for over a thousand years [8]. Wenxin Keli (WXKL), a classical Chinese patent medicine with high efficacy and favorable safety, is useful for the management of patients with AF [9–11]. WXKL is composed of five Chinese herbal extracts: Radix (Dang Shen, DS), Polygonati Rhizoma (Huang Jing, HJ), Notoginseng Radix et Rhizoma (San Qi, SQ), Ambrum (Hu Po, HP), and Nardostachyos Radix et Rhizoma (Gan Song, GS). It is the first Chinese antiarrhythmic medicine approved by the China Food and Drug Administration [11–13].

Recent animal and cell studies [14–16] have demonstrated that WXKL inhibits and prevents atrial arrhythmias via complicated antiarrhythmic mechanisms, but the specific molecular mechanism remains unclear.

**Figure 1:**
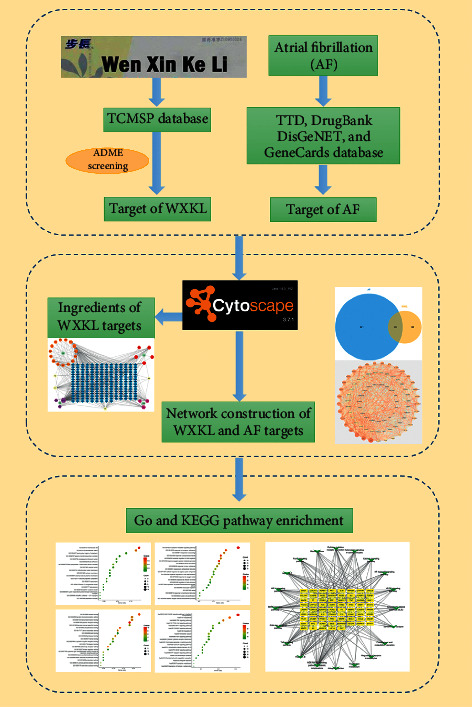
Analysis framework based on an integrated network pharmacology strategy.

Similar to other TCM formulas, WXKL is a multicomponent and multitarget agent that achieves its specific therapeutic efficacy via the regulation of molecular networks by active components. In this study, we used a comprehensive network pharmacology-based approach to investigate the mechanisms by which WXKL exerts therapeutic effects in AF. A flowchart of the experimental procedure is shown in Figure 1.

## 2. Materials and Methods

### 2.1. Database Building and Active Compound Screening

All compounds in the five herbal components of WXKL were retrieved from the TCM Systems Pharmacology (TCMSP, http://lsp.nwu.edu.cn/tcmsp.php) database and analysis platform, which captures relationships among drugs, targets, and diseases. The database includes chemicals, targets, drug-target networks, and associated drug-target-disease networks, as well as pharmacokinetic properties of natural compounds, such as oral bioavailability (OB), drug-likeness (DL), and blood-brain barrier penetration [17, 18]. Compounds were screened based on absorption, distribution, metabolism, and excretion (ADME), and pharmacokinetic information retrieval filters were used to obtain bioactive compounds for further analysis using the thresholds OB ≥ 30% and DL ≥ 0.18 [19, 20]. A network was generated using Cytoscape to visualize the complex relationships between active compounds and potential targets [21]. Nodes represent compounds and targets, and edges indicate the intermolecular interactions.

### 2.2. Identification of Drug Targets

Protein targets of the compounds were retrieved from the TCMSP. Gene names were extracted from UniProtKB (http://www.uniprot.org).

### 2.3. Screening of Potential Targets for AF

AF-associated target genes were obtained from various databases, such as TTD, DrugBank, DisGeNET, and GeneCards. The DrugBank database (https://www.drugbank.ca/) is a unique bioinformatics and cheminformatics resource that combines detailed drug data with comprehensive drug target information [22, 23]. The DisGeNET database (https://www.disgenet.org/) includes one of the largest publicly available collections of genes and variants associated with human diseases [24]. The GeneCards database (https://www.genecards.org/) is a searchable, integrative database that provides comprehensive, user-friendly information for all annotated and predicted human genes [25]. The term “atrial fibrillation” was used for searches against these four databases to screen targets related to AF.

### 2.4. Collection of Common Compound-Disease Targets

The screened chemical targets and disease targets were imported into the ImageGP (http://www.ehbio.com/ImageGP/index.php) platform, and common compound-disease targets were obtained as candidates for further analysis [26].

### 2.5. PPI Network of Compound-Disease Targets

A protein-protein interaction (PPI) network was generated using the STRING database (https://string-db.org/), which covered almost all functional interactions between the expressed proteins [27]. Species was set to “Homo sapiens,” and the target interaction information was obtained. The results were imported into Cytoscape (version 3.6.1; https://www.cytoscape.org/) to draw and analyze the interaction network. The node size reflected the number of combined edges (degree), and nodes with a degree greater than twice the median degree of all nodes were selected as hubs.

### 2.6. Gene Ontology (GO) and KEGG Pathway Enrichment Analyses

GO analyses based on the three main categories, i.e., biological process (BP), molecular function (MF), and cell component (CC), and a Kyoto Encyclopedia of Genes and Genomes (KEGGs) (https://www.kegg.jp/) pathway enrichment analysis were performed using the Metascape system (http://metascape.org/gp/index.html) [28] and the ImageGP. A *P* value less than 0.05 indicated significance.

### 2.7. Pathway Constructions and Analysis

In order to explore the biological effects of cellular targets affecting the diseases through modulating specific pathways, an incorporated “AF pathway” was integrated based on the current understanding of AF pathology [29]. In brief, the obtained target proteins were firstly mapped to KEGG to distribute them to several pathways. Next, pathways closely related to AF were picked out and consolidated into an “AF pathway” under the pathological and clinical data.

## 3. Results

### 3.1. Identification of Active Compounds in WXGs

From the TCMSP, 354 total compounds were retrieved, including 134 in Radix (DS), 38 in Rhizoma (HJ), 119 in Notoginseng Radix et Rhizoma (SQ), 6 in Ambrum (HP), and 57 in Nardostachyos Radix et Rhizoma (GS). Among the 354 compounds, 34 satisfied OB ≥ 30% and DL ≥ 0.18. Finally, 30 compounds were obtained after excluding duplicates (Table 1).

### 3.2. WXKL Target Identification

**Figure 2:**
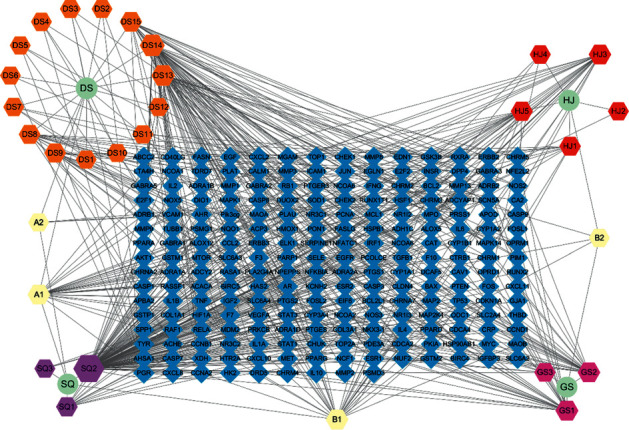
Drug-compound-target network of potential targets in WXKL. Blue diamond represents potential active ingredients in WXKL, compounds in WXKL are shown in light yellow, pink, red, orange, and purple, and the herbs are shown in green. Radix (Dang Shen, DS), Polygonati Rhizoma (Huang Jing, HJ), Notoginseng Radix et Rhizoma (San Qi, SQ), Ambrum (Hu Po, HP), and Nardostachyos Radix et Rhizoma (Gan Song, GS). A1 represents stigmasterol. A2 represents Diop. B1 represents beta-sitosterol. B2 represents sitosterol. DS1 represents daturilin. DS2 represents (8 S, 9 S, 10 R, 13 R, 14 S, 17 R)-17-[(E, 2 R, 5 S)-5-ethyl-6-methylhept-3-en-2-yl]-10,13-dimethyl-1,2,4,7,8,9,11,12,14,15,16,17-dodecahydrocyclopenta[a]phenanthren-3-one. DS3 represents ZINC03978781. DS4 represents poriferasta-7,22E-dien-3beta-ol. DS5 represents spinasterol. DS6 represents stigmast-7-enol. DS7 represents 11-hydroxyrankinidine. DS8 represents 3-beta-hydroxymethyllenetanshiquinone. DS9 represents frutinone A. DS10 represents 7-(beta-xylosyl)cephalomannine_qt. DS11 represents perlolyrine. DS12 represents methyl icosa-11,14-dienoate. DS13 represents 7-methoxy-2-methyl isoflavone. DS14 represents luteolin. DS15 represents glycitein. GS1 represents cryptotanshinone. GS2 represents acacetin. GS3 represents (2R)-5,7-dihydroxy-2-(4-hydroxyphenyl)chroman-4-one. HJ1 represents diosgenin. HJ2 represents (+)-syringaresinol-O-beta-D-glucoside. HJ3 represents baicalein. HJ4 represents 4′,5-dihydroxyflavone. HJ5 represents 3′-methoxydaidzein. SQ1 represents ginsenoside rh2. SQ2 represents quercetin. SQ3 represents mandenol.

In total, 30 compounds in WXKL were associated with 645 target proteins. After eliminating overlapping proteins, 219 proteins were obtained. To further understand the interactions between compounds and targets, we constructed a compound-target network, as shown in Figure 2, by mapping 30 compounds to 219 potential targets associated with inflammation, antioxidant stress, nuclear factor-kappa B, immune regulation, and so on. The network had 253 nodes and 531 edges. Compounds with the most targets in WXKL were quercetin, luteolin, 7-methoxy-2-methyl isoflavone, beta-sitosterol, baicalein, and stigmasterol, with 141, 55, 40, 35, 31, and 28 targets, respectively. These results suggested that the six components are probably related to the therapeutic effects in AF.

### 3.3. Retrieval of Potential Disease Targets

**Figure 3:**
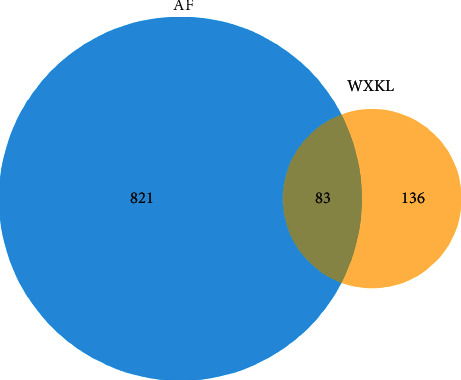
Matching of target genes for AF and WXKL.

TTD, DrugBank, DisGeNET, and GeneCards retrieval results were integrated to obtain AF-related disease targets. As shown in Figure 3, the potential targets of WXKL were mapped to the disease targets using the ImageGP platform, and a Venn diagram was drawn. A total of 83 potential targets were obtained based on the intersection of the two sets of targets. These targets are summarized in Supplementary Table S1.

### 3.4. Analyses of Candidate Target Proteins

**Figure 4:**
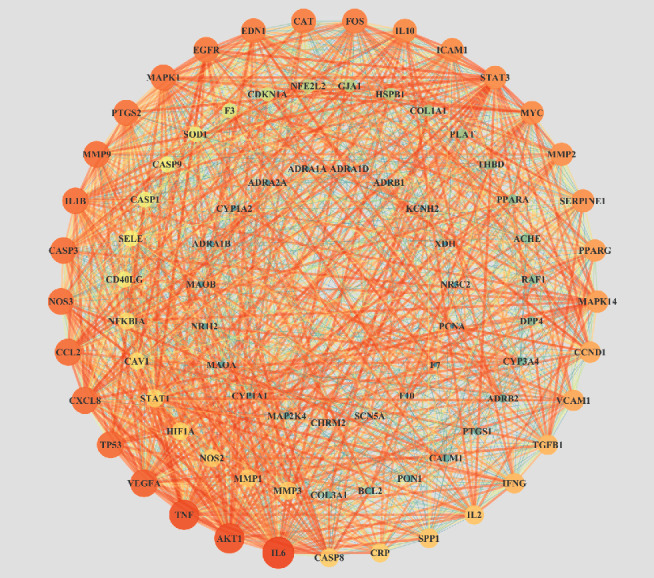
PPI network of targets for WXKL in the treatment of AF. Layout of the three rings corresponds to the area and color of nodes. Nodes represent potential targets of WXKL in AF. Node size from large to small indicates a decrease in the degree value. Lines connecting inner nodes indicate relationships between proteins.

A total of 83 potential genes associated with AF were uploaded to the STRING database for analysis. The systematically selected protein targets with a median confidence score of 0.400 were plotted as a PPI network using the STRING database. A total of 83 nodes and 1253 edges were acquired, and the average node degree was 30.2. Furthermore, using Cytoscape, we constructed a network (Figure 4), in which the edges represent associations between a pair of targets, nodes represent the target, and the degree value represents the intensity. The top ten targets IL-6, TNF, AKT1, VEGFA, CXCL8, TP53, CCL2, MMP9, CASP3, and NOS3 had high degrees in this process, explaining their significance in the network.

### 3.5. Gene Ontology Enrichment Analysis

We imported the 83 candidate target genes into the Metascape system for a GO analysis.

**Figure 5:**
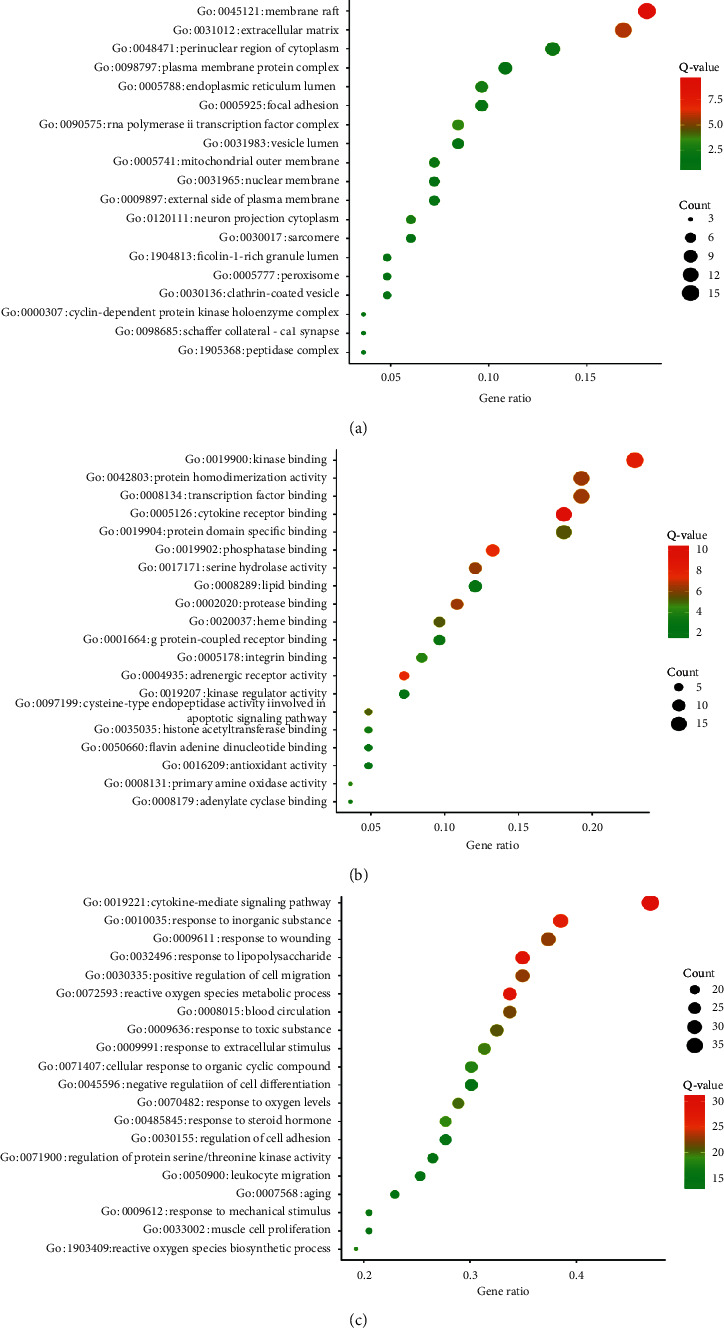
GO enrichment analyses of potential targets of the main active ingredients of WXKL: (a) cell component (CC); (b) molecular function (MF); (c) biological process (BP). Node colors are shown as a gradient from red to green according to the descending order of *P* values. Node sizes are in the ascending order of the gene count.

Based on this analysis, the potential targets were related to many biological processes, molecular functions, and cellular components, including functions that maybe important for the occurrence and development of AF. The top 20 highly enriched BP terms were retained for analysis and included negative regulation of cytokine, reactive oxygen species, and cell migration (Figure 5(c)). Previous study showed that AF targets were the diversity of molecular pathways affecting mechanical stress, sarcomere disarray, chronic inflammation, reactive oxygen species, and activation of atrial fibroblasts, which have collectively emerged from translational research and genome-wide association studies (GWASs) [30]. These results indicated the therapeutic effects of WXKL in AF maybe mediated by its effects on these biological processes. Accordingly, these processes are of great significance for our understanding of the mechanism by which WXKL influences AF.

A total of 119 GO terms in the molecular function category (Figure 5(b)) were enriched, and the top 20 entries were selected for analysis. These terms mainly included cytokine receptor binding, kinase binding, adrenergic receptor activity, phosphatase binding, and antioxidant activity.

In total, 58 GO terms in the cell components category (Figure 5(a)) were enriched, and the top 19 entries were selected for analysis based on *P* < 0.01. The targets were closely related to membrane raft extracellular matrix, RNA polymerase II transcription factor complex, endoplasmic reticulum lumen, neuron projection cytoplasm, mitochondrial outer membrane, perinuclear region of cytoplasm, focal adhesion, and nuclear membrane. Previous study showed interconnectivity of numerous targets functioning in the cardiomyocyte cell membrane, sarcoplasmic reticulum, nucleus, sarcomere, and inflammasome with reported roles in human or preclinical models of AF [31, 32]. Thus, it can be seen that WXKL plays a role in the treatment of AF through these cell components category.

### 3.6. KEGG Pathway Enrichment Analysis

**Figure 6:**
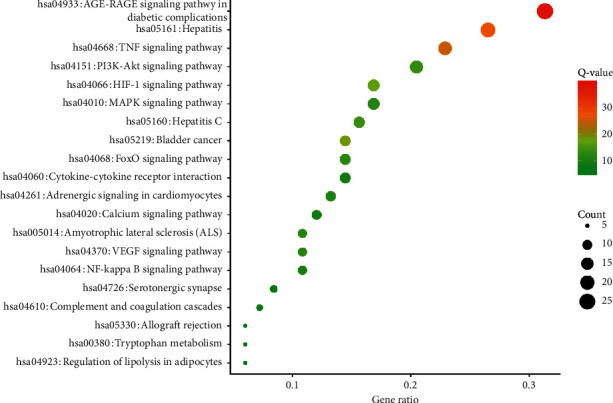
KEGG pathway enrichment analyses of potential targets of the main active ingredients of WXKL. Node colors are shown as a gradient from red to green according to the descending order of *P* values. Node sizes are in the ascending order of the gene count.

To further reveal the mechanisms underlying the effect of WXKL on AF, we conducted a KEGG pathway enrichment analysis of 83 targets and screened the top 20 pathways based on a threshold value of *P* < 0.01 (Figure 6). Many pathways involving potential target genes were identified, such as the VEGF signaling pathway, HIF-1 signaling pathway, and PI3K-Akt signaling pathway, which are associated with signal transduction. Hepatitis B, TNF signaling pathways, NF-kappa B signaling, cytokine-cytokine receptor interaction, and hepatitis C pathways are related to inflammatory reactions. Moreover, amyotrophic lateral sclerosis, allograft rejection, and allograft rejection are closely related to immunological processes. The calcium signaling pathway modulates intracellular Ca^2+^ levels and a number of Ca^2+^-dependent intracellular signaling processes. Serotonergic synapses and tryptophan modulate 5-hydroxytryptamine, which is a hormonal trigger for AF. In addition, complement and coagulation cascades revealed that WXKL has a potential role in other related diseases. The AGE-RAGE signaling pathway is highly important in diabetic complications; it elicits the activation of multiple intracellular signaling pathways involving NADPH oxidase, protein kinase C, and MAPKs, thereby resulting in NF-kappa B activity.

Previous study showed that the mechanisms of AF were related to atrial cardiomyopathy, mechanical stress, activation of atrial fibroblasts, fibrofatty infiltrations, inflammation, activation of inflammasomes and macrophages, hypercoagulability and autonomic nervous system dysregulation, sarcoplasmic reticulum Ca^2+^ leak, and so on [30].

**Figure 7:**
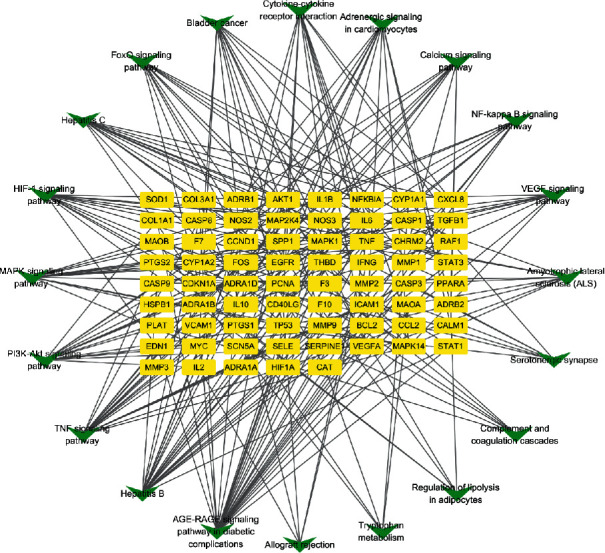
Target-pathway network for WXKL. Green and yellow nodes represent pathways and targets, respectively, and edges represent interactions.

**Figure 8:**
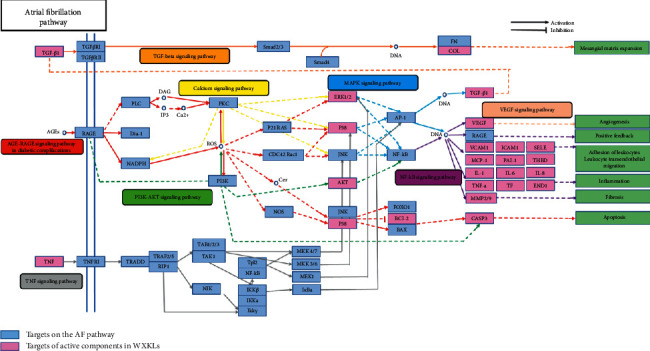
Atrial fibrillation pathway and therapeutic modules.

The results verified that WXKL alleviates AF by regulating inflammatory, immunity, antioxidant stress, serotonergic synapses, Ca^2+^ levels, and a number of Ca^2+^-dependent intracellular signaling processes (Figures 7 and 8).

## 4. Discussion

AF, the most common sustained arrhythmia, currently affects over 33 million individuals worldwide, and its prevalence is expected to be more than double over the next 40 years. AF is associated with a twofold increase in premature mortality and important major adverse cardiovascular events, such as heart failure, severe stroke, and myocardial infarction [33]. AF is not just an atrial disease, with documented associations with systemic inflammation, endothelial dysfunction, cardiometabolic disturbance, and wider abnormalities in myocardial structure and function [34, 35]. In this study, we identified the main active ingredients of WXKL as quercetin, luteolin, 7-methoxy-2-methyl isoflavone, beta-sitosterol, baicalein, and stigmasterol and used network and functional analyses to show that they are potentially valuable for the treatment of AF.

Some compounds detected in this study have reported effects other than anti-inflammatory, antiimmune, and antistress activity. Quercetin, a very common flavonoid found in plant foods, prevents arrhythmias and dramatically improves heart muscle function and repair following heart attack. Quercetin has a protective effect against AF, probably by inhibiting platelet aggregation and TXA2 formation, as well as increasing PGI2 generation [35]. Previous studies have suggested that luteolin, a flavonoid, possesses antioxidative, antitumor, and anti-inflammatory properties. The cardiac protective effects of luteolin have recently been demonstrated in vitro and in vivo [36].

As illustrated in the compound-target network, many targets could be altered by multiple compounds, such as TNF, IL-6, JUN, and PTGS1. Others, including CRP, CXCL2, and COL3A1, could only be regulated by quercetin. These results indicated that WXKL has multicomponent and multitarget biological characteristics. A PPI network showed that WXKL could regulate the expression of AF-regulated targets and alleviate AF symptoms. IL-6 (degree = 65), AKT1 (degree = 61), TNF (degree = 61), VEGFA (degree = 56), and TP53 (degree = 53) are potential hub targets in the network.

To predict the mechanisms underlying the therapeutic effects of WXKL in AF, we evaluated the candidate targets by GO enrichment analyses. Based on the top 20 GO terms (*P* < 0.01) in each major category, the major hubs were significantly enriched for multiple biological processes, including negative regulation of cytokine, reactive oxygen species, and cell migration, as shown in Figure 5(c). Furthermore, they were enriched for various molecular functions, including cytokine receptor binding, kinase binding, adrenergic receptor activity, phosphatase binding, and antioxidant activity, as shown in Figure 5(b). The active targets IL-6, AKT1, TNF, CCL18, CCL2, and NOS3 were related to various molecular processes, particularly immune regulation, oxidative stress, and inflammatory response. To some extent, these results are consistent with the pathogenesis and clinical features of AF. The key cellular components included membrane raft extracellular matrix, RNA polymerase II transcription factor complex, endoplasmic reticulum lumen, neuron projection cytoplasm, mitochondrial outer membrane, perinuclear region of cytoplasm, focal adhesion, vesicle lumen, cyclin-dependent protein kinase holoenzyme complex, and nuclear membrane, and many targets were highly ranked as potential-related genes. These findings indirectly illustrated the complexity of the pathogenesis of AF and the damage to various cellular components.

It is generally believed that oxidative stress, inflammation, and related processes are important factors for the promotion of apoptosis or myocardial fibrosis, leading to atrial electrical remodeling and structural remodeling and eventually to the occurrence and persistence of AF [37, 38]. The identification of significant leukocyte infiltration in atrial myocardium in AF patients and observed correlation between elevated circulating proinflammatory cytokines and AF severity support this contention [39]. Established inflammatory factors and biomarkers related to AF include tumor necrosis factor *α*, C-reactive protein, interleukin-2, interleukin-6, interleukin-8, matrix metalloproteinase, endothelin, and myeloperoxidase [34]. Cardiac fibroblasts which were a highly plastic population of resident cells play an important role in the development of an AF substrate [40]. Activated fibroblasts secrete extracellular matrix proteins and recruit immune cells, leading to dispersion and blockade of conduction in the myocardium. Combined with an already heterogeneous and anisotropic fiber bundle arrangement, focal fibrosis renders the left atrium extremely susceptible to reentrant circuits [40, 41]. VEGF, a pivotal activator of angiogenesis and calcium (Ca^2+^) signaling in endothelial cells, increases collagen production in atrial fibroblasts [42]. In addition, after treatment, procollagen type I, procollagen type III production, myofibroblast differentiation, and the migratory ability of fibroblasts are reduced in AF. These results further confirm that the targets identified in this study are consistent with previous reports, indicating supporting the therapeutic effects of WXKL.

Obesity is associated with an increased amount of epicardial fat, which is a major mediator of the relationship between obesity and AF [43]. A probable mechanism by which epicardial fat promotes AF is direct adipocyte infiltration into the atrial myocardium, resulting in slow or anisotropic conduction. A second probable mechanism is atrial fibrosis caused by adipokines secreted from epicardial fat. Third, it is possible that epicardial fat contributes to AF by the secretion of proinflammatory factors, such as IL-6, IL-8, and TNF-*α*. By a KEGG analysis, we found that WXKL can regulate lipolysis in adipocytes (Figures 7 and 8), implying that it is a good treatment strategy for patients with obesity and AF.

In 2019, Wang et al. [44] proposed that the relationship between AF and DM is complex and is mediated by structural, electrical, electromechanical, and autonomic changes in the atria, triggered by oxidative stress, inflammation, and glycemic fluctuations. Glucose-lowering therapies may affect the development of AF. Accordingly, it is important to elucidate the mechanism underlying DM-related AF and to evaluate the best treatment strategy for patients with DM and AF. Our KEGG analysis indicated that the most important pathway by which WXKL effects AF is the AGE-RAGE signaling pathway (Figures 7 and 8), involved in diabetic complications, which elicits the activation of multiple intracellular signaling pathways involving NADPH oxidase, protein kinase C, and MAPKs, resulting in NF-kappa B activity. Combining our KEGG analysis, WXKL maybe is a good treatment strategy for patients with DM and AF.

Serotonin, 5-hydroxytryptamine, is a hormonal trigger for AF [45, 46]. By a KEGG analysis, we found that pathways are related to the role of WXKL in AF including the regulation of serotonergic synapses and tryptophan metabolism.

Accordingly, the targets identified in our study are consistent with previous findings, further supporting the use of WXKL for the treatment of AF by regulating the inflammatory response, oxidative stress reaction, immune regulation, cardiac energy metabolism, tryptophan metabolism, and other pathways.

## 5. Conclusions

In summary, WXKL is beneficial for the treatment of AF, consistent with previous studies. We further characterized the biological functions of active ingredients in WXKL and their corresponding targets by a network pharmacological approach, improving our understanding of the molecular biological mechanism underlying the effects of WXKL in AF. These findings provide an important theoretical basis for the clinical treatment of AF.

## Figures and Tables

**Table 1:** Basic information for components of WXKL.

MOL ID	Molecule name	OB (%)	DL
MOL008397	Daturilin	50.37	0.77
MOL008393	7-(Beta-xylosyl)cephalomannine_qt	38.33	0.29
MOL007514	Methyl icosa-11,14-dienoate	39.67	0.23
MOL009763	(+)-Syringaresinol-O-beta-D-glucoside	43.35	0.77
MOL008407	(8 S, 9 S, 10 R, 13 R, 14 S, 17 R)-17-[(E, 2 R, 5 S)-5-Ethyl-6-methylhept-3-en-2-yl]-10,13-dimethyl-1,2,4,7,8,9,11,12,14,15,16,17-dodecahydrocyclopenta[a]phenanthren-3-one	45.4	0.76
MOL006774	Stigmast-7-enol	37.42	0.75
MOL003036	ZINC03978781	43.83	0.76
MOL001006	Poriferasta-7,22E-dien-3-beta-ol	42.98	0.76
MOL004355	Spinasterol	42.98	0.76
MOL002879	Diop	43.59	0.39
MOL000359	Sitosterol	36.91	0.75
MOL001494	Mandenol	42	0.19
MOL008411	11-Hydroxyrankinidine	40	0.66
MOL002140	Perlolyrine	65.95	0.27
MOL006331	4′,5-Dihydroxyflavone	48.55	0.19
MOL001040	(2R)-5,7-Dihydroxy-2-(4-hydroxyphenyl)chroman-4-one	42.36	0.21
MOL005344	Ginsenoside rh2	36.32	0.56
MOL005321	Frutinone A	65.9	0.34
MOL000546	Diosgenin	80.88	0.81
MOL007059	3-Beta-hydroxymethyllenetanshiquinone	32.16	0.41
MOL002959	3′-Methoxydaidzein	48.57	0.24
MOL008400	Glycitein	50.48	0.24
MOL001689	Acacetin	34.97	0.24
MOL007088	Cryptotanshinone	52.34	0.4
MOL000449	Stigmasterol	43.83	0.76
MOL002714	Baicalein	33.52	0.21
MOL000358	Beta-sitosterol	36.91	0.75
MOL003896	7-Methoxy-2-methyl isoflavone	42.56	0.2
MOL000006	Luteolin	36.16	0.25
MOL000098	Quercetin	46.43	0.28

## Data Availability

The data used to support the findings of this study are included within the article.

## Supplementary Materials

Supplementary Materials See Table S1 in the Supplementary Material for information on potential targets and topological attributes.

## References

[1] Sumeet S., Chugh R., Havmoeller K. (2014). Worldwide epidemiology of atrial fibrillation: a global burden of disease 2010 study.. *Circulation*.

[2] Wolf P. A., Abbott R. D., Kannel W. B. (1991). Atrial fibrillation as an independent risk factor for stroke: the framingham Study. *Stroke*.

[3] Gómez-Outes A., Lagunar-Ruíz J., Terleira-Fernández A.-I., Calvo-Rojas G., Suárez-Gea M. L., Vargas-Castrillón E. (2016). Causes of death in anticoagulated patients with atrial fibrillation. *Journal of the American College of Cardiology*.

[4] Chung M. K., Eckhardt L. L., Chen L. Y. (2020). Lifestyle and risk factor modification for reduction of atrial fibrillation: a scientific statement from the American heart association. *Circulation*.

[5] Verma A., Jiang C., Betts T. R. (2015). Approaches to catheter ablation for persistent atrial fibrillation. *New England Journal of Medicine*.

[6] Upadhyay G. A., Alenghat F. J. (2019). Catheter ablation for atrial fibrillation in 2019. *JAMA*.

[7] Spitzer S. G., Károlyi L., Rämmler C. (2017). Treatment of recurrent nonparoxysmal atrial fibrillation using focal impulse and rotor mapping (FIRM)-Guided rotor ablation: early recurrence and long-term outcomes. *Journal of Cardiovascular Electrophysiology*.

[8] Wang Z., Tang W. Z., GeGe J. L. (2017). Efficacy and safety of traditional Chinese medicine on thromboembolic events in patients with atrial fibrillation: a systematic review and meta-analysis. *Complementary Therapies in Medicine*.

[9] Zhuogen H., Minan Z., Pingchang X., Yuanping W., Xia Y., Dingwei D. (2018). Wenxin Keli for atrial fibrillation. *Medicine*.

[10] Zhang N., Tse S. G., GongYang M. Y. (2018). Efficacy of wenxin keli plus amiodarone versus amiodarone monotherapy in treating recent-onset atrial fibrillation. *Cardiology Research and Practice*.

[11] Chan G., Sun Y., Liu S. (2018). Therapeutic effects of wenxin keli in cardiovascular diseases: an experimental and mechanism overview. *Frontiers in Pharmacology*.

[12] Rui Z., Guihua T., Qin Z. (2018). Clinical safety and efficacy of wenxin keli-amiodarone combination on heart failure complicated by ventricular arrhythmia: a systematic review and meta-analysis. *Frontiers in Physiology*.

[13] Liu Y., Zhang Z., Yang Y. N., Li G., Liu T. (2016). The Chinese herb extract Wenxin Keli: a promising agent for the management of atrial fibrillation. *International Journal of Cardiology*.

[14] Zhang J.-W., Li W., Guo K. (2016). Antiarrhythmic effects and potential mechanism of WenXin KeLi in cardiac purkinje cells. *Heart Rhythm*.

[15] Chen Y., Yang L., Guo L. (2013). Effects of wenxin keli on the action potential and L-type calcium current in rats with transverse aortic constriction-induced heart failure. *Evidence-Based Complementary and Alternative Medicine*.

[16] Minoura Y., Panama B. K., Nesterenko V. V. (2013). “Effect of Wenxin Keli and quinidine to suppress arrhythmogenesis in an experimental model of Brugada syndrome. *Heart Rhythm*.

[17] Betzenhauser J., Peng L., Wang J. (2014). TCMSP: a database of systems pharmacology for drug discovery from herbal medicines. *Journal of Cheminformatics*.

[18] Zhu B., Zhang W., Lu Y. (2018). Network pharmacology-based identification of protective mechanism of Panax Notoginseng Saponins on aspirin induced gastrointestinal injury. *Biomedicine & Pharmacotherapy*.

[19] Xu X.-x., Bi J.-p., Ping L., Li P., Li F. (2018). A network pharmacology approach to determine the synergetic mechanisms of herb couple for treating rheumatic arthritis. *Drug Design, Development and Therapy*.

[20] Guo M.-F., Dai Y. J., Jia-Rong G. (2020). Uncovering the mechanism of Astragalus membranaceus in the treatment of diabetic nephropathy based on network pharmacology. *Journal of Diabetes Research*.

[21] Qin T., Wu L., Hua Q., Song Z., Pan Y., Liu T. (2020). Prediction of the mechanisms of action of Shenkang in chronic kidney disease: a network pharmacology study and experimental validation. *Journal of Ethnopharmacology*.

[22] Wang Y., Zhang S., Li F. (2020). Therapeutic target database 2020: enriched resource for facilitating research and early development of targeted therapeutics. *Nucleic Acids Research*.

[23] Wishart D. S., Feunang Y. D., Guo A. C. (2018). DrugBank 5.0: a major update to the DrugBank database for 2018. *Nucleic Acids Research*.

[24] Piñero J., Ramírez-Anguita J. M., Saüch-Pitarch J. (2020). The DisGeNET knowledge platform for disease genomics: 2019 update. *Nucleic Acids Research*.

[25] Stelzer G., Rosen R., Plaschkes I. (2016). The GeneCards suite: from gene data mining to disease genome sequence analysis.. *Curr Protoc Bioinformatics*.

[26] Zeng L. Y., Wang Q., Han L. Y. (2019). Mechanism of tripterygium hypoglaucum-leonuri Herba for treating rheumatoid arthritis based on network pharmacology. *Chinese Journal of Experimental Traditional Medical Formulae*.

[27] Szklarczyk D., Gable A. L., Lyon D. (2019). STRING v11: protein-protein association networks with increased coverage, supporting functional discovery in genome-wide experimental datasets. *Nucleic Acids Research*.

[28] Zhou Y., Zhou B., Pache L. (2019). Metascape provides a biologist-oriented resource for the analysis of systems-level datasets. *Nature Communications*.

[29] Liu J., Mu J., Zheng C. (2016). Systems-pharmacology dissection of traditional Chinese medicine compound saffron formula reveals multi-scale treatment strategy for cardiovascular diseases. *Entific Reports*.

[30] Ang Y.-S., Rajamani S., Haldar S. M., Huser J. (2020). A new therapeutic framework for atrial fibrillation drug development. *Circulation Research*.

[31] Hüser N., Bianca J. J. M. B. (2020). Inflammasomes and proteostasis novel molecular mechanisms associated with atrial fibrillation. *Circulation Research*.

[32] Nattel S., Heijman J., Zhou L., Dobromir D. (2020). “Molecular basis of atrial fibrillation pathophysiology and therapy. *Circulation Research*.

[33] Dobrev R. S., Casadei B. (2019). Mechanisms of atrial fibrillation. *Heart*.

[34] Wijesurendra R. S., Casadei B. (2015). Atrial fibrillation: effects beyond the atrium?. *Cardiovascular Research*.

[35] Xiao D., Gu Z. L., Qian Z. N. (1993). Effects of quercetin on platelet and reperfusion-induced arrhythmias in rats. *Acta Pharmacologica Sinica*.

[36] Luo Y., Shang P., Li D. (2017). Luteolin: a flavonoid that has multiple cardio-protective effects and its molecular mechanisms.. *Frontiers in Pharmacology*.

[37] Li J., Solus J., Chen Q. (2010). Role of inflammation and oxidative stress in atrial fibrillation. *Heart Rhythm*.

[38] Hadi H. A. 1, Alsheikh-Ali A. A., WA M., Jassim M. A. S. (2010). Inflammatory cytokines and atrial fibrillation: current and prospective views. *Journal of Inflammation Research*.

[39] Schotten U., Verheule S., Kirchhof P., Goette A. (2011). “Pathophysiological mechanisms of atrial fibrillation: a translational appraisal. *Physiological Reviews*.

[40] Ho S. Y., Cabrera J. A., Damian S. Q. (2012). Left atrial anatomy revisited. *Circulation: Arrhythmia and Electrophysiology*.

[41] Burstein B., Libby E., Nattel S. (2008). Differential behaviors of atrial versus ventricular fibroblasts. *Circulation*.

[42] Chung C.-C., Lin Y.-K., Chen Y.-C., Kao Y.-H., Lee T.-I., Chen Y.-J. (2020). Vascular endothelial growth factor enhances profibrotic activities through modulation of calcium homeostasis in human atrial fibroblasts. *Laboratory Investigation*.

[43] Wong C. X., Ganesan A. N., Selvanayagam J. B (2017). Epicardial fat and atrial fibrillation: current evidence, potential mechanisms, clinical implications, and future directions. *European Heart Journal*.

[44] Wang A., Green J. B., Halperin J. L., Piccini J. P. (2019). Atrial fibrillation and diabetes mellitus. *Journal of the American College of Cardiology*.

[45] Yusuf S., Al-Saady N., Camm A. J. (2003). 5-Hydroxytryptamine and atrial fibrillation. *Journal of Cardiovascular Electrophysiology*.

[46] Murray K. T., Mace L. C., Yang Z. (2007). Nonantiarrhythmic drug therapy for atrial fibrillation. *Heart Rhythm*.

