# Supplementary material for: Molecular Targets and Pathways Contributing to the Effects of Wenxin Keli on Atrial Fibrillation Based on a Network Pharmacology Approach
Source: Evid Based Complement Alternat Med. 2020 Oct 7;2020:8396484. doi: 10.1155/2020/8396484 (PMC7586041; doi:10.1155/2020/8396484)
Supplement: Supplementary Materials — See Table S1 in the Supplementary Material for information on potential targets and topological attributes. [file 8396484.f1.docx]

**Supplementary Table S1: Information on potential targets and the topological attributes.**

| No. | Gene name | Protein name | UniProt ID | Degree |
| --- | --- | --- | --- | --- |
| 1 | IL6 | Interleukin-6 | P05231 | 65 |
| 2 | AKT1 | RAC-alpha serine/threonine-protein kinase | P31749 | 61 |
| 3 | TNF | Tumor necrosis factor | P01375 | 61 |
| 4 | VEGFA | Vascular endothelial growth factor A | P15692 | 56 |
| 5 | TP53 | Cellular tumor antigen p53 | P04637 | 54 |
| 6 | CXCL8 | Interleukin-8 | P10145 | 54 |
| 7 | CCL2 | C-C motif chemokine 2 | P13500 | 54 |
| 8 | NOS3 | Nitric oxide synthase, endothelial, EC 1.14.13.39 | P29474 | 53 |
| 9 | CASP3 | Caspase-3 | P42574 | 53 |
| 10 | IL1B | Interleukin-1 beta | P01584 | 53 |
| 11 | MMP9 | Matrix metalloproteinase-9 | P14780 | 53 |
| 12 | MAPK1 | Mitogen-activated protein kinase 1 | P28482 | 52 |
| 13 | PTGS2 | Prostaglandin G/H synthase 2 | P35354 | 52 |
| 14 | EGFR | Epidermal growth factor receptor | P00533 | 51 |
| 15 | CAT | Catalase | P04040 | 50 |
| 16 | FOS | Proto-oncogene c-Fos | P01100 | 50 |
| 17 | EDN1 | Endothelin-1 | P05305 | 50 |
| 18 | IL10 | Interleukin-10 | P22301 | 48 |
| 19 | STAT3 | Signal transducer and activator of transcription 3 | P40763 | 47 |
| 20 | MYC | Myc proto-oncogene protein | P01106 | 47 |
| 21 | ICAM1 | Intercellular adhesion molecule 1 | P05362 | 47 |
| 22 | SERPINE1 | Plasminogen activator inhibitor 1 | P05121 | 46 |
| 23 | MMP2 | 72 kDa type IV collagenase | P08253 | 46 |
| 24 | MAPK14 | Mitogen-activated protein kinase 14 | Q16539 | 44 |
| 25 | PPARG | Peroxisome proliferator-activated receptor gamma | P37231 | 44 |
| 26 | CCND1 | G1/S-specific cyclin-D1 | P24385 | 42 |
| 27 | VCAM1 | Vascular cell adhesion protein 1 | P19320 | 42 |
| 28 | TGFB1 | Transforming growth factor beta-1 proprotein | P01137 | 40 |
| 29 | IFNG | Interferon gamma | P01579 | 40 |
| 30 | IL2 | Interleukin-2 | P60568 | 38 |
| 31 | SPP1 | Osteopontin | P10451 | 38 |
| 32 | CASP8 | Caspase-8 | Q14790 | 37 |
| 33 | CRP | C-reactive protein | P02741 | 37 |
| 34 | NOS2 | Nitric oxide synthase, inducible, EC 1.14.13.39 | P35228 | 35 |
| 35 | MMP3 | Stromelysin-1 | P08254 | 35 |
| 36 | MMP1 | Interstitial collagenase | P03956 | 35 |
| 37 | HIF1A | Hypoxia-inducible factor 1-alpha | Q16665 | 34 |
| 38 | STAT1 | Signal transducer and activator of transcription 1-alpha/beta | P42224 | 34 |
| 39 | CAV1 | Caveolin-1 | Q03135 | 33 |
| 40 | NFKBIA | NF-kappa-B inhibitor alpha | P25963 | 33 |
| 41 | CD40LG | CD40 ligand | P29965 | 31 |
| 42 | SELE | E-selectin | P16583 | 31 |
| 43 | CASP1 | Caspase-1 | P29466 | 30 |
| 44 | CASP9 | Caspase-9 | P55211 | 30 |
| 45 | SOD1 | Superoxide dismutase [Cu-Zn], EC 1.15.1.1 | P00441 | 29 |
| 46 | F3 | Tissue factor | P13726 | 28 |
| 47 | CDKN1A | Cyclin-dependent kinase inhibitor 1 | P38936 | 27 |
| 48 | NFE2L2 | Nuclear factor erythroid 2-related factor 2 | Q16236 | 25 |
| 49 | HSPB1 | Heat shock protein beta-1 | P04792 | 24 |
| 50 | GJA1 | Gap junction alpha-1 protein | P17302 | 24 |
| 51 | COL1A1 | Collagen alpha-1(I) chain | P02452 | 22 |
| 52 | PLAT | Tissue-type plasminogen activator | P00750 | 21 |
| 53 | THBD | Thrombomodulin, TM | P07204 | 20 |
| 54 | PPARA | Peroxisome proliferator-activated receptor alpha | Q07869 | 19 |
| 55 | ACHE | Acetylcholinesterase | P22303 | 19 |
| 56 | RAF1 | RAF proto-oncogene serine/threonine-protein kinase | P04049 | 18 |
| 57 | DPP4 | Dipeptidyl peptidase 4 | P27487 | 17 |
| 58 | CYP3A4 | Cytochrome P450 3A4 | P08684 | 16 |
| 59 | ADRB2 | Beta-2 adrenergic receptor | P07550 | 15 |
| 60 | CALM1 | Calmodulin-1 | P0DP23 | 14 |
| 61 | PTGS1 | Prostaglandin G/H synthase 1 | P23219 | 14 |
| 62 | BCL2 | Apoptosis regulator Bcl-2 | P10415 | 13 |
| 63 | COL3A1 | Collagen alpha-1(III) chain | P02461 | 13 |
| 64 | PON1 | Serum paraoxonase/arylesterase 1 | P27169 | 13 |
| 65 | MAP2K4 | Dual specificity mitogen-activated protein kinase kinase 4 | P45985 | 12 |
| 66 | CYP1A1 | Cytochrome P450 1A1 | P04798 | 11 |
| 67 | MAOA | Amine oxidase | P21397 | 11 |
| 68 | NR1I2 | Nuclear receptor subfamily 1 group I member 2 | O75469 | 10 |
| 69 | MAOB | Amine oxidase [flavin-containing] B | P27338 | 10 |
| 70 | ADRA1B | Alpha-1B adrenergic receptor | P35368 | 9 |
| 71 | CYP1A2 | Cytochrome P450 1A2 | P05117 | 9 |
| 72 | ADRA2A | Alpha-2A adrenergic receptor | P08913 | 9 |
| 73 | ADRA1A | Alpha-1A adrenergic receptor | P35348 | 8 |
| 74 | ADRA1D | Alpha-1D adrenergic receptor | P25100 | 8 |
| 75 | ADRB1 | Alpha-1B adrenergic receptor | P35368 | 8 |
| 76 | PCNA | Proliferating cell nuclear antigen | P12004 | 6 |
| 77 | F7 | Coagulation factor VII, EC 3.4.21.21 | P08709 | 6 |
| 78 | F10 | Coagulation factor X | P00742 | 6 |
| 79 | KCNH2 | Potassium voltage-gated channel subfamily H member 2 | Q12809 | 6 |
| 80 | XDH | Xanthine dehydrogenase/oxidase | P47989 | 6 |
| 81 | NR3C2 | Mineralocorticoid receptor | P08235 | 6 |
| 82 | SCN5A | Sodium channel protein type 5 subunit alpha | Q14524 | 4 |
| 83 | CHRM2 | Muscarinic acetylcholine receptor M2 | P08172 | 4 |
